# The Future of (Soil) Microbiome Studies: Current Limitations, Integration, and Perspectives

**DOI:** 10.1128/mSystems.00613-21

**Published:** 2021-08-24

**Authors:** Stefan Geisen

**Affiliations:** a Laboratory of Nematology, Wageningen University, Wageningen, The Netherlands

**Keywords:** microbiome, food webs, protists, soil microbiology, trophic interactions

## Abstract

Microbes dominate the planet’s biodiversity in terms of species number and by driving essential Earth system functions such as the carbon cycle. Soils contain most of this microbial biodiversity. Only recently, we have started to better understand the diversity of bacteria and fungi at the global scale. Here, I list my views on some shortcomings of contemporary soil microbiome studies and potential solutions to overcome them. In particular, I highlight that (soil) microbiome studies should become more holistic in terms of (i) taxa and resolution targeted, (ii) by adding functional to taxonomic information, and (iii) by integrating temporal analysis into spatial analyses. Considering those elements with the methodology that is now available will advance our understanding on (soil) microbiomes to reliably address major ecological hypotheses and to advance insights into the importance for life on Earth.

## COMMENTARY

What is life on the planet? In the general public, most people consider biodiversity to be represented mainly by plants and animals, with the remaining biodiversity mostly being treated as maleficent pathogens. Indeed, the past period has been shaped by the coronavirus disease 2019 (COVID-19) pandemic. Beyond viruses, also other microbial groups, including bacteria, fungi, and protists, are mainly known as pathogens of humans, animals, or plants. These organisms are of direct relevance for humanity and together with the fact that targeted studies can easily focus on these individual pathogenic species explain the major interest on pathogens. While microbial biodiversity has a century long history that started with the invention of the microscope, microbial ecology has experienced a major burst in research especially in the last decade. Sequencing techniques have shifted the previously often individual-based, pathogen-centered approaches to untargeted community-level analyses that do not require visible host symptoms (1). Starting with host-associated microbiome studies, microorganisms are now studied in all imaginable systems, including soils (2).

Still, a bias remains toward microbial studies in hosts. This contrasts with the actual diversity of microbes in different systems, which in host-associated systems is by far lower than in the environment (3). It has become evident that soils contain the highest diversity, abundance, and biomass of microbes (4, 5). Most research in hosts and soils is also not equally spread among microbial groups but focused predominantly on bacteria and to some extend fungi (6). Among the major achievements were knowledge gains on different spatial patterns, including global distribution based on surrounding physicochemical properties (7, 8), but increasing evidence hints to the importance of biotic interactions in driving bacterial and fungal communities, such as competition (9) and especially top-down predation (10). Yet, knowledge on microbiome predators strongly lags behind knowledge on their microbial prey.

## A PARADIGM SHIFT TOWARDS INTEGRATED MICROBIOME STUDIES

I strongly believe that most knowledge gains in the field of soil biology can be obtained by more integrative studies that include a range of aspects that I will compile in the following sections.

## THE BENEFIT OF REAL MICROBIOME STUDIES BEYOND THE FOCUS ON BACTERIAL COMMUNITIES

**FIG 1:**
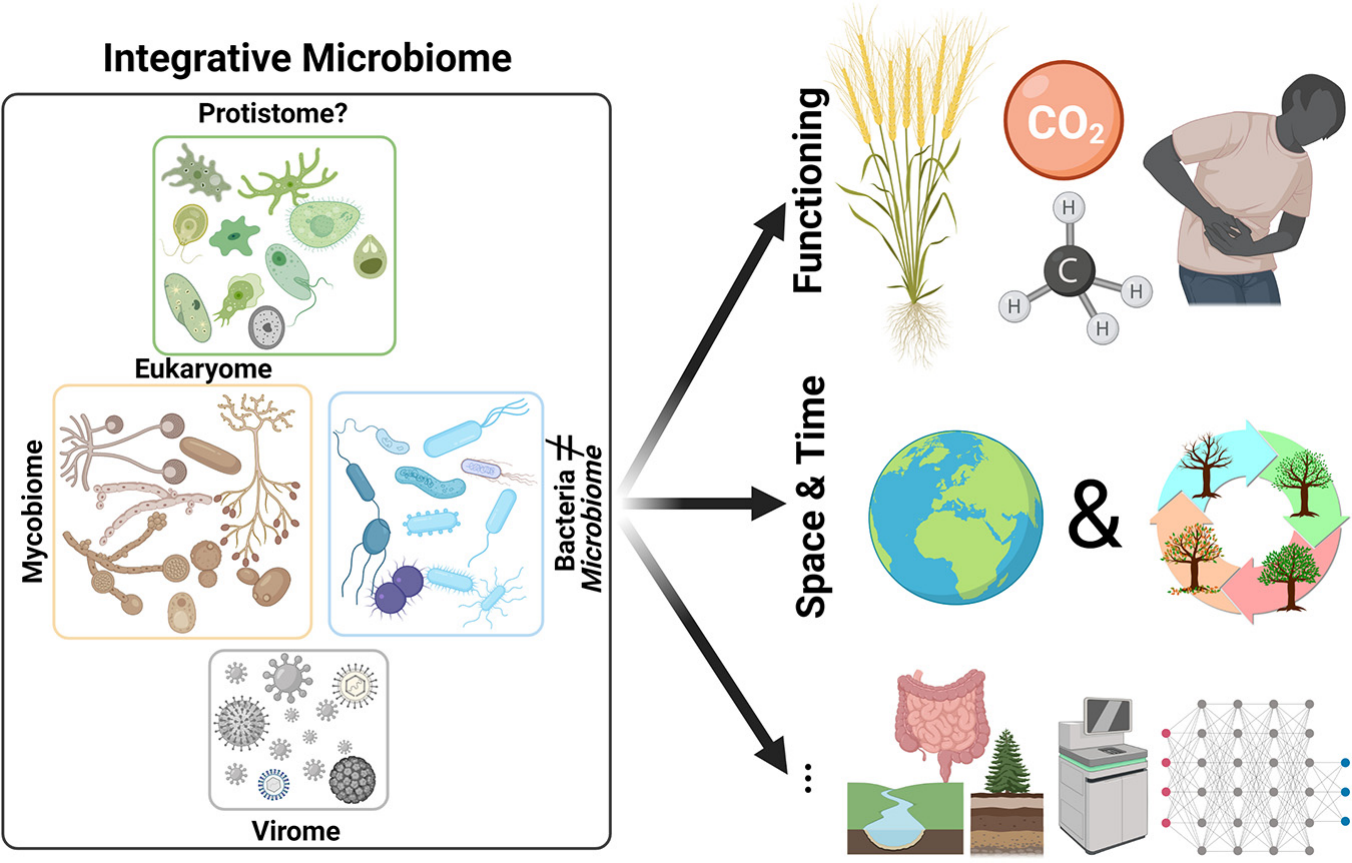
Overview of envisioned microbiome work that profoundly steps beyond the current focus mostly on describing bacterial communities. I envision integrative microbiome studies to cover different microbial groups (left). Furthermore, added value will come from linking the microbiome to its function such as its effect on plant growth, greenhouse gas emissions, or host performance (top right), from environmental studies both through space and time (middle right), and from other advances such as cross-system comparisons, using novel methods or statistical approaches (bottom right). The figure was created with BioRender.com.

Currently, most “microbiome” studies study only bacteria and thereby use the term microbiome synonymously for bacteria. As such, alternative terms for other microbial groups have evolved; these terms include mycobiome to focus on fungi (11), virome to focus on viruses (12), and eukaryome to focus on microbial eukaryotes (13). While there is some use of these terms, the many omes arguably represent buzzwords that could endlessly be broken down to smaller and smaller units. I propose using microbiome only if at least several microbial groups are studied simultaneously, ideally all. In fact, arguably in almost any environment from the gut to the deep sea to Antarctic soils, some bacterial interactions with other microbiome groups occur, such as competition with fungi, infection with viruses, or predator-prey interactions with protists. At least the presence of other microbiome groups should be investigated before a study centered on bacteria claims to be a true microbiome study (Fig. 1).

There are more reasons for integrated taxon approaches in microbiomics than semantics: microbial groups interact and shape another often in ways comparable to abiotic factors. Indeed, competition between fungi and bacteria shape their communities (9), while predation by protists and lysis by viruses accelerate turnover and community composition of bacteria (10, 14). Beyond these direct interactions, integrative microbiome studies have the advantage of detecting environmental differences that might not be visible for one group of microbes. For example, we revealed that protists showed severe community fluctuations over time and due to fertilization, whereas communities of bacteria and fungi did not vary (15). We also revealed that bacterial and especially protistan communities and protistan species predict plant health unlike fungal communities (16). Therefore, microbiome studies should include multiple microbial groups, as otherwise misleading conclusions on bacterial community assembly, ecological patterns, or treatment effect sizes might be drawn.

Last, all large-scale microbiome studies in soils and even hosts have a major disadvantage: they focus on community metrics that deviate from biogeographic studies on individual species-based studies of plants and animals. Given these limitations, how can we target basic ecological questions and real species-level biogeography and even monitor potential biodiversity declines such as those evident from macroscopic plants and animals (17)? Furthermore, species-level analyses are crucial to finally increase the known biodiversity of species in soils that remain below 10% (18). We now have the tools available to make a step forward from the mostly performed short-read community-based sequencing profiles to tools that allow differentiation of individual microbial species, such as long-read sequencing tools such as PacBio, Nanopore, or LoopSeq (19, 20).

## MICROBIOMES NEED FUNCTIONAL INFORMATION

Much knowledge gains on the biogeographic distribution of microbial groups, particularly bacteria and fungi, have been obtained at different spatial scales up to the global level (2, 7–9). More recently, protists and nematodes have also been added to the list of globally studied minute organisms (4, 21, 22). As such, we now understand the basic physicochemical properties that determine microbial biogeography. Yet, we have now reached a point where pure diversity comparisons have little additive value, and we need to acknowledge and better study the immense functional importance of microbes (Fig. 1). Tools to assign functions from taxonomic identities can be a start for conserved functions and include programs such as PICRUSt2 and Tax4Fun (23) for bacteria, FUNGuild (24) and FungalTraits (25) for fungi, and NINJA for nematodes (26).

However, DNA-based efforts can distort the real functioning of organisms in the environment as inactive organisms are part of the recovered diversity. Furthermore, taxonomy-based functional annotations can be misleading (e.g., soil bacteria do not necessarily function as related gut bacteria; fungi with identical marker gene sequences can range from pathogenic to mutualistic). The next steps in functional studies are omics approaches that provide direct information on functional genes and actively transcribed genes (27). Classical cultivation-based interaction studies are also experiencing a comeback, as they are used to provide ultimate evidence for the functioning of individual microorganisms alone and in combination. This fact is evidenced by increasing efforts in cultivation techniques (28) that will further possibilities for functional assays. These include phytometer-based studies to investigate microbial effects on plant growth and classical (soil) biological methods like greenhouse gas measurements and functional gene measurements. Generally, more blended studies that include multiple experimental and analytical approaches are ideal to provide a more cumulative understanding of microbial functioning; these approaches include the ones above at different scale (e.g., field to greenhouse to laboratory), from molecular to biochemical ones (e.g., metagenomics, metatranscriptomics, metaproteomics, metabolomics, and stable-isotope-based methods), and analytical approaches (e.g., indicator analyses, network analyses, and structural equation). Obviously, cost constraints do not allow the application of all methods simultaneously nor would they always make sense to include. I am instead proposing tailored method combinations to back up some of the often standardly performed sequence-based approaches, which should be defined based on the research question and also in terms of availability and cost-effectiveness. I believe that everyone that can do deep metagenomics can afford some additional functional experiments or analyses even if that would mean to sequence less deeply.

## MICROBIOME STUDIES NEED BOTH A SPATIAL AND TEMPORAL DIMENSION

As introduced above, we have gained a thorough understanding of the global biogeography of microbes, but all these studies have one major drawback: all contemporary large-scale soil biodiversity inventories have been obtained at a single point in time, whereas local-scale studies revealed the importance of seasonal factors in shaping soil biodiversity (29). These temporal changes are predictable, as constant fluctuations in physicochemical factors, such as moisture, temperature, or the availability of different carbon and nutrient substrates, largely determine the global distribution of soil biodiversity (7, 8, 21, 22). For example, considering that protists are mostly determined by soil moisture (21) and that protists determine the community, abundance, and functioning of bacteria and fungi (10), short-term temporal variations in precipitation might cause serious shifts in the soil microbiome. Also, the functioning of soil biodiversity can hardly be extrapolated from single time point sampling schemes especially in nontropical regions, as microbial activity is strongly dependent on temperature. How can we then predict general microbial communities and their function from a single time point while constant changes are expected? To increase the meaningfulness of large-scale surveys in terms of microbial communities and their functioning, we need a better understanding of temporal changes through multiple intra- and interannual sampling approaches (Fig. 1).

## MY VISION ON FUTURE MICROBIOME STUDIES

Above, I listed some of the options to increase the meaningfulness and scope of microbiome research, with many other potential improvements available. These include methodological improvements and integration, links between systems, many of which are listed by G. Berg et al. (6). What is really needed in (soil) microbiome studies? Considering all these aspects simultaneously (Fig. 1)! Obviously, this is not the task of individual studies, but more to give researchers a better framework for all the aspects that exist in microbiome research. Furthermore, the above points should help to better and more reliably interpret observed patterns in distinct microbiome studies, as all multidimensional aspects around the field of microbiomes can never be targeted in individual research.

In my ongoing and future research, I will try to increase the knowledge on different aspects on (soil) microbiomes with a focus on aspects that remain hardly known, such as including diverse microbial groups at a taxonomic resolution of species ideally, add multiple functional information to taxon-based ones, and include a temporal axis to most microbiome studies. My vision is that other researchers also aim at expanding their research interest to step away from the standard analyses that seem to be inherent to many current microbiome analyses, i.e., sequencing a short barcoding region of the 16S rRNA gene at different spatial scales or systems, analyze these data with the same bioinformatic pipelines and R scripts to eventually publish very similar papers—with differences mainly in systems studied or increased analyses based on the same data. Obviously, this point of view is highly provocative and does not capture many ongoing highly innovative studies. I am convinced that by incorporating some of the approaches mentioned above, microbiome research will become more honest, holistic, integrative, and meaningful.
